# Supplementary material for: Extensive sequencing of seven human genomes to characterize benchmark reference materials
Source: Sci Data. 2016 Jun 7;3:160025. doi: 10.1038/sdata.2016.25 (PMC4896128; doi:10.1038/sdata.2016.25)
Supplement: Supplementary Information [file sdata201625-s2.doc]

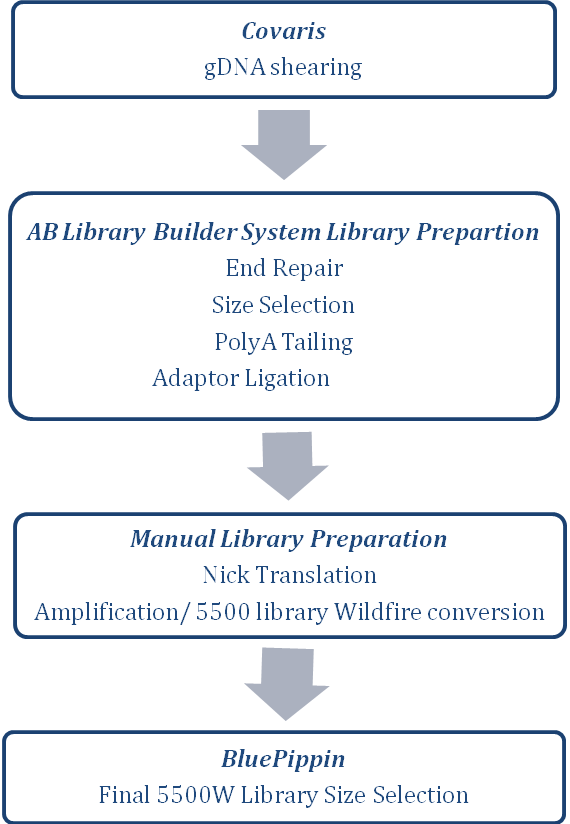


Supplementary Figure 1: Workflow to produce libraries for 5500W sequencing of HG-005


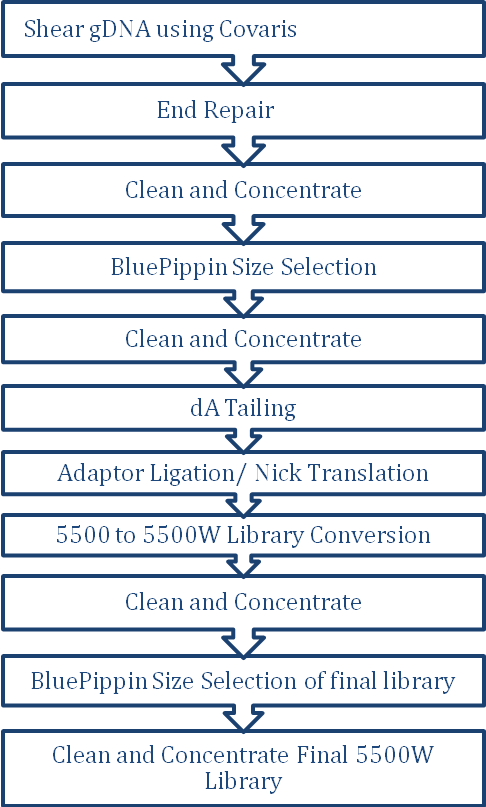


Supplementary Figure 2: Workflow to produce libraries for 5500W sequencing of HG-002


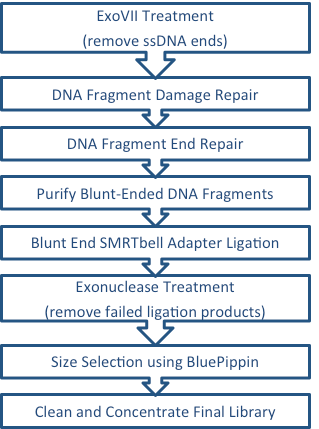


Supplementary Figure 3: Workflow to produce libraries for Pacific Biosciences sequencing
